# Patients with polyclonal hepatocellular carcinoma are at a high risk of early recurrence and have a poor recurrence-free survival period

**DOI:** 10.1007/s12072-021-10278-4

**Published:** 2022-01-01

**Authors:** Masaki Kaibori, Kazuko Sakai, Hideyuki Matsushima, Hisashi Kosaka, Kosuke Matsui, Marco A. De Velasco, Mitsugu Sekimoto, Kazuto Nishio

**Affiliations:** 1 Department of Surgery, Hirakata Hospital, Kansai Medical University, Hirakata, Osaka Japan; 2 Department of Genome Biology, Faculty of Medicine, Kindai University, 377-2 Ohno-Higashi, Osaka-Sayama, Osaka 589-8511 Japan

**Keywords:** Clonal composition, Hepatocellular carcinoma, Recurrence-free survival, Copy number variation, Gene expression, Gene set enrichment analysis, Early recurrence, Single nucleotide polymorphism array, B-allele frequency, Proliferation

## Abstract

**Background/purpose of the study:**

Tumor heterogeneity based on copy number variations is associated with the evolution of cancer and its clinical grade. Clonal composition (CC) represents the number of clones based on the distribution of B-allele frequency (BAF) obtained from a genome-wide single nucleotide polymorphism (SNP) array. A higher CC number represents a high degree of heterogeneity. We hypothesized and evaluated that the CC number in hepatocellular carcinoma (HCC) tissues might be associated with the clinical outcomes of patients.

**Methods:**

Somatic mutation, whole transcriptome, and CC number based on copy number variations of 36 frozen tissue samples of operably resected HCC tissues were analyzed by targeted deep sequencing, transcriptome analysis, and SNP array.

**Results:**

The samples were classified into the heterogeneous tumors as poly-CC (*n* = 26) and the homogeneous tumors as mono-CC (*n* = 8). The patients with poly-CC had a higher rate of early recurrence and a significantly shorter recurrence-free survival period than the mono-CC patients (7.0 months vs. not reached, *p* = 0.0084). No differences in pathogenic non-synonymous mutations, such as *TP53*, were observed between the two groups when targeted deep sequencing was applied. A transcriptome analysis showed that cell cycle-related pathways were enriched in the poly-CC tumors, compared to the mono-CC tumors. Poly-CC HCC is highly proliferative and has a high risk of early recurrence.

**Conclusion:**

CC is a possible candidate biomarker for predicting the risk of early postoperative recurrence and warrants further investigation.

**Supplementary Information:**

The online version contains supplementary material available at 10.1007/s12072-021-10278-4.

## Introduction

Liver cancer is one of the most common malignancies, with more than 800,000 new cases diagnosed globally each year [1]. Hepatocellular carcinoma (HCC) accounts for approximately 75% of liver cancers. Although surgical resection improves the survival of HCC patients, early recurrence after hepatic resection is a poor prognostic factor for patients with HCC. HCC is a highly heterogeneous cancer [2–4]. Molecular heterogeneity and a lack of biomarkers for recurrence have contributed to the poor prognosis of HCC patients.

The intratumoral heterogeneity of cancer cells remains largely unexplored. Copy number variations reflect genomic structural changes that give rise to gene amplification or deletion. Chromosomal microarray and other array-based approaches have been widely adopted for the detection of whole-genome copy number variations [5, 6]. The OncoScan FFPE Assay Kit (Thermo Fisher Scientific, Wilmington, DE) relies on molecular inversion prove (MIP) technology to detect genome-wide copy number alterations, loss of heterozygosity, and somatic mutation [7]. This assay provides the B-allele frequency (BAF), the log2 ratio (log2R), and the copy number for each of over 220,000 analyzed polymorphic genomic locations. The copy number is derived from the log2R and BAF values. The clonal composition of a tumor can be analyzed based on the BAF and log2R, which can be determined from the whole-genome copy number profiles obtained using the OncoScan FFPE Assay Kit. We previously demonstrated that the whole-genome single nucleotide polymorphism (SNP) array could be applied to the detection of the clonal composition of human ovarian cancer. Genome-wide segmentation data consisting of the log2R and BAF have been previously used to estimate the clonal composition (CC) number for individual tumors [8]. A similar approach resulted in a report indicating that the clonal composition can be derived from both somatic mutations as well as the log2R and BAF for loci with an aberrant copy number [9]. The CC number is not equivalent to the number of clones in the tumor tissue, but instead represents and estimates the number of clones that show copy number changes. For instance, a BAF value of 0.5 indicates a heterozygous genotype (AB), whereas values of 0 and 1 indicate homozygous genotypes (AA and BB, respectively). The BAF distribution in segments with copy number changes suggests the presence of different clones with different BAF patterns intratumorally. In segments with copy number gains, the percentage of aberrant cells with different BAF patterns due to BAF variation is difficult to evaluate because the copy number gain is not constant. On the other hand, there is no such limitation in evaluating the BAF in segments with copy number loss, and the clonal composition number can be derived from the presence of aberrant cells with a different BAF pattern.

We hypothesized that HCC with a high CC would exhibit highly aggressive behaviors when compared with HCC with a low CC. We examined the CC numbers of previously collected frozen tissue samples of HCC and investigated the association with early recurrence in postoperative HCC patients. In addition, we also used NGS-based deep sequencing to detect somatic mutations and to examine the whole transcriptome of HCC tissues to investigate any associations with CC.

## Materials and methods

### Hepatocellular carcinoma samples

Frozen tumor tissue samples were obtained from 36 patients who underwent surgical resection at Kansai Medical University Hospital between August 2011 and December 2013. All the patients provided written informed consent to participate in the study, including the collection of tumor frozen tissues for analysis. Tumor stage is defined by General Rules for the Clinical and Pathological Study of Primary Liver Cancer [10] as stage I–IVa. All cases including five cases of IVa tumors were completely resected. The early recurrence of postoperative HCC has been previously defined as recurrence within 1 year of resection [11, 12]. The recurrence-free survival (RFS) and overall survival (OS) were defined as the time interval between the date of hepatectomy and the date of recurrence or death, respectively. After surgery, patients were followed in the outpatient clinic every 3 months for the first 2 years and every 6 months thereafter. Early recurrence was defined as the appearance of a new lesion compatible with HCC on radiologic examination during the follow-up period.

### Isolation of genomic DNA and RNA

A 0.5 cm square tumor legion without necrosis visually was sectioned and used for the analysis. DNA and RNA were isolated from frozen tissues using an AllPrep DNA/RNA Mini Kit (Qiagen, Valencia, CA) according to the manufacturer's instructions. The quality and quantity of the nucleic acid were verified using a NanoDrop 2000 device, PicoGreen dsDNA Reagent, and RiboGreen RNA reagent (all from Thermo Scientific).

### Microarray-based comparative genomic hybridization assay

A MIPs array for the genome-wide estimation of copy number aberrations was performed using the OncoScan FFPE Assay Kit (Thermo Fisher Scientific), as previously reported [8]. Briefly, 80 ng of DNA was subjected to annealing with these MIPs for 16–18 h, followed by enzyme digestion and two separate gap-fill reactions. The circular MIPs were then separately linearized for each gap fill with a cleavage enzyme and amplified using PCR. The PCR products were subjected to enzymatic cleavage and fragmentation, followed by hybridization for 16–18 h with two OncoScan arrays (one for each gap fill). The arrays were then stained and washed using a GeneChip Fluidics Station 450 and loaded into a GeneChip Scanner 3000 7G (Affymetrix). Array fluorescence intensity (CEL) files were generated using Affymetrix GeneChip Command Console (AGCC) software, version 4.0, and the CEL files were converted to OSCHP files using OncoScan Console software 1.3 and visualized with Chromosome Analyses Suite software (version 4.0). A representative whole-genome view is shown in Fig. S1.

### Clonal composition analysis

Clonal composition numbers were calculated using BAF and log2R information obtained from the OncoScan FFPE Assay using the Onco Clone Composition program, as described previously [8]. A given copy number segment can be associated with a percentage of aberrant tumor cells (%AC) and is assumed to result from a single underlying event attributable to a single clone; in other words, the copy number segments are associated with the same %AC and belong to the same clone. The clonal composition number is calculated by identifying the number of different %AC detected among the aberrant segments. The number of minor alleles (NOMA) at a heterozygous site can be defined as the BAF of a segment. The calculation of clone composition number only uses NOMA = 0, which represents a segment with 100% loss of heterozygosity and a normal copy number. If only one of such alleles is present, the BAF for such a segment will be zero. The Onco Clone Composition program provides analytical estimations of clonal composition using a clustering approach that clusters all segments with the same logR and BAF and then combines clusters corresponding to the same %AC. A graphical representation of the aberrated segments and their association with %AC is shown in Supplementary Fig. S2.

### Targeted DNA sequencing

A targeted DNA library comprising approximately 1.2-Mb of the coding regions of 409 genes for panel sequencing was constructed using an Ion AmpliSeq Comprehensive Cancer Panel (CCP) (Thermo Fisher Scientific), as described previously [13]. Briefly, 40 ng of DNA was subjected to multiplex PCR amplification using an Ion AmpliSeq Library Kit 2.0 and the Ion AmpliSeq Comprehensive Cancer Panel (Thermo Fisher Scientific), which covers all exons in 409 genes. After multiplex PCR, Ion Xpress Barcode Adapters (Thermo Fisher Scientific) were ligated to the PCR products, which were then purified using Agencourt AMPure XP beads (Beckman Coulter, Brea, CA). The purified libraries were pooled and then sequenced using an Ion Torrent S5 instrument and the Ion 550 Chip Kit (all from Thermo Fisher Scientific). DNA sequencing data were accessed through the Torrent Suite ver. 5.10 program (Thermo Fisher Scientific). Reads were aligned against the hg19 human reference genome, and variants were called using Variant Caller, ver. 5.10. Raw variant calls were filtered with a quality score of < 100 and were manually checked using the integrative genomics viewer (IGV; Broad Institute, Cambridge, MA). Germline mutations were excluded using the Human Genetic Variation Database (http://www.genome.med.kyoto-u.ac.jp/SnpDB) and the Exome Aggregation Consortium database. Variants with FATHMM scores greater than 0.7 were classified as pathogenic [14]. The tumor mutation burden (TMB mutations/Mb) was assessed using Ion Reporter Software, ver. 5.10 (Thermo Fisher Scientific).

### Whole transcriptome analysis

The whole transcriptome analysis was performed using the AmpliSeq Transcriptome Human Gene Expression Kit (Thermo Fisher Scientific). For library preparation, cDNA was generated using the SuperScript VILO cDNA Synthesis kit (Thermo Fisher Scientific) from 10 ng of total RNA. Then, cDNA was amplified for 12 cycles by adding PCR Master Mix and the AmpliSeq human transcriptome gene expression primer pool (Thermo Fisher Scientific). After multiplex PCR, Ion Xpress Barcode Adapters (Thermo Fisher Scientific) were ligated to the PCR products, which were then purified using Agencourt AMPure XP beads (Beckman Coulter). The purified libraries were pooled and then sequenced using an Ion Torrent S5 instrument and the Ion 550 Chip Kit (all from Thermo Fisher Scientific). The Ion Torrent Suite v5.10 software (Thermo Fisher Scientific) was used for base calling, alignment to the human reference genome (hg19), and quality control. Raw reads were then analyzed automatically using the AmpliSeqRNA plugin to generate gene-level expression values for all 20,802 RefSeq human genes.

### Gene set enrichment analysis (GSEA)

A gene-set enrichment analysis (GSEA) was performed to identify pathways enriched in the Molecular Signatures Database (MSigDB) Hallmark gene set [15, 16]. A nominal *p* value of < 0.05 and an FDR (false discovery rate) *q* value of < 0.05 were considered statistically significant.

### Gene selection and pathway analysis

A total of 2408 genes that were differentially expressed according to CC numbers were selected using the following criteria: *p* value < 0.05 and FDR *q* value < 0.5. Among these 2408 genes, the top 500 markers of differential expression were identified based on the signal-to-noise ratio. Hierarchical clustering was performed using one minus the Pearson correlation coefficient as a distance measure and the average linkage method. To explore the potential biological pathways, genes in a cluster were submitted to the Metascape tool (https://metascape.org/gp/index.html) [17].

### Tumor infiltration analysis

Tumor IMmune Estimation Resource (TIMER2.0, http://timer.comp-genomics.org/) was used to estimate immune infiltration levels of immune cells including B cell, CD4+ T cell, CD8+ T cell, Neutrophil, Macrophage, and Myeloid dendritic cell [18–20]. The gene expression data from whole transcriptome analysis was normalized as RPM (reads per million mapped reads).

### Immunohistochemical staining

A standard immunohistochemistry analysis was performed for the cytokeratin 19 (CK19) and Epithelial cell adhesion molecule (EpCAM) as follows. Formalin-fixed tissue sections were sectioned and placed on positively charged slides and pretreated with steam heating in DAKO target retrieval solution (Catalog #S1699, Agilent, Santa Clara, CA) for 20 min. Slides were incubated with anti-CK19 antibody (Catalog #13092, Cell Signaling, Danvers, MA, dilution 1:100) and anti-EpCAM antibody (Catalog #14452, Cell Signaling, dilution 1:100), overnight stained using the ABC kit (Vector Laboratories, Burlingame, CA) following the manufacturer’s protocols, developed in diaminobenzidine DAB (Invitrogen, Carlsbad, CA), and counterstained with hematoxylin. Assessment of immunohistochemical staining was performed on scanned colon mucosa sections captured at 10 × magnification. H scores were calculated for CK19 or EpCAM positivity in the cancerous regions using QuPath v0.3.0-m4 image analysis software (https://qupath.github.io/).

### Statistical analysis

Categorical variables were compared using the Fisher exact test. Continuous variables were compared between groups with the Mann–Whitney *U* test. Variables with a statistical significance in the univariate analysis were included in the multiple logistic regression analysis. Survival was estimated using the Kaplan–Meier method and the log-rank test method. All the statistical analyses were performed using JMP software, version 14.2 (SAS Institute, Cary, NC), and Prism software, version 8.4 (GraphPad Software, San Diego, CA). A *p* value of < 0.05 was considered statistically significant.

## Results

### Clinicopathological characteristics of patients

Clinicopathological characteristics of patients are shown in Table 1. The median age was 71.5 years (range 50–87), and 22 patients (61.1%) were male gender. Thirty-three patients (91.7%) were Child–Pugh class A. Patients with HBV and HCV infection were 7 (19.4%) and 14 (38.9%), respectively. The median ALT level was 29.5 U/l (range 9–92), total bilirubin level was 0.7 mg/dl (range 0.3–1.6), the median albumin level was 4.15 g/dl (range 2.6–5), median AFP level was 37.6 ng/ml (range 2–261,351), and the median PIVKA-II level was 93 mAU/ml (range 13–75,000). The median ICGR15 value was 12.9% (range 2.3–50.1). Seven patients received preoperative treatment included monotherapy of transcatheter arterial chemoembolization (TACE, *n* = 5), combination therapy of TACE and percutaneous transhepatic portal vein embolization (PTPE, *n* = 1), combination therapy of TACE and radiofrequency ablation (RFA, *n* = 1). All patients with stage I–IVa received curable resection, and the maximum tumor diameter was 4 cm (range 0.8–140). The number of tumors ranged from 1 to 3. Patients classified into well (8/36, 22.2%), moderately (24/36, 66.7%), and poor (2/36, 5.6%) differentiated HCC. Microscopic vascular invasion was noted in 83.3% (30/36) of patients. Early recurrence within 12 months after curative resection occurred in 22 patients (61.1%). All patients with recurrence had intrahepatic recurrence. Distant metastases were found in the lung (1 patient) and left adrenal gland (1 patient).

**Table 1:** Patients characteristics

Variable	Category	*n* = 36
Sex	Male/Female	22/14 (61.1/38.9)
Age	Median (range)	71.5 (50–87)
Liver function status	Child–Pugh A/B	33/3 (91.7/8.3)
Etiology	HBV/HCV/alcoholic/normal	7/14/2/13 (19.4/38.9/5.6/36.1)
ALT (U/l)	Median (range)	29.5 (9–92)
Total bilirubin (mg/dl)	Median (range)	0.7 (0.3–1.6)
Albumin (g/dl)	Median (range)	4.15 (2.6–5)
ICGR15 (%)	Median (range)	12.9 (2.3–50.1)
AFP (ng/ml)^a^	Median (range)	37.6 (2–261,351)
PIVKA-II (mAU/ml)^a^	Median (range)	93 (13–75,000)
Tumor size (cm)^a^	Median (range)	4 (0.8–140)
Tumor number	Median (range)	1 (1–3)
Microscopic vascular invasion	Positive/negative	30/6 (83.3/16.7)
Histologic grade	well/moderate/poor/unknown	8/24/2/2 (22.2/66.7/5.6/5.6)
Stage^a^	I/II/III/IVa	1/8/21/5 (2.9/22.9/60.0/14.3)
History of previous treatment	Present/absent	7/29 (19.4/80.6)
Early recurrence	Yes/no	22/14 (61.1/38.9)

The asterisk indicates statistical significance

^a^Missing data in one patient

### Clonal composition number of resectable hepatocellular cancer

We analyzed 36 frozen samples of HCC using a whole-genome SNP array (Fig. S1) and estimated the clonal composition number using the Onco Clone Composition estimation program (Fig. S2). The mean ± SD value for the CC number was 1.0 ± 0.8 (range 0–3). It should be noted that a case with a CC number of 0 indicates a homogenous tumor mass. The CC number could not be estimated in two cases because the copy number data did not plot onto the fitting curve in the program. Representative plots for the CC profile are shown in Fig. S2. The HCC samples were categorized into mono-CC (CC = 0, *n* = 8) and poly-CC (CC ≥ 1, *n* = 26) groups that respectively reflected homogenous and heterogenous clones.

### Clinicopathological features associated with clonal composition status

**Fig. 1:**
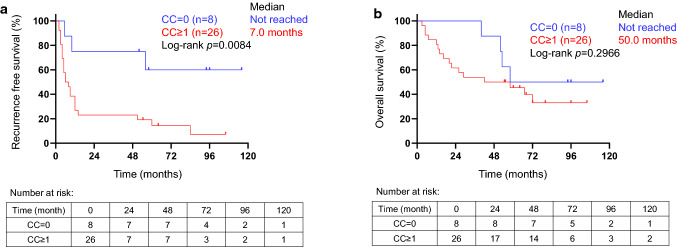
Kaplan–Meier curves of recurrence-free survival (RFS) (**a**) and overall survival (OS) (**b**) for patients with mono-CC (CC = 0) (blue line) and poly-CC (CC ≥ 1) (red line)

The clinicopathological characteristics of patients with mono-CC or poly-CC are summarized in Table 2. No significant differences were observed in sex, mean age, liver function status (Child–Pugh A or B), or stage as determined using the Fisher exact test. The presence of microscopic vascular invasion (*p* = 0.0180), moderately to poorly differentiated HCC (*p* = 0.0469), absence of neoadjuvant therapy (*p* = 0.0374), the presence of single tumor nodule (*p* = 0.0374), and higher levels of PIVKA-II (*p* = 0.0351) were significantly frequent in poly-CC group. This result suggests that poly-CC shows an aggressive tumor phenotype compared to mono-CC group. Early recurrence was significantly frequent in the poly-CC group than in the mono-CC group (*p* = 0.0127). There was no difference in the incidence of extrahepatic metastasis between poly-CC and mono-CC. The RFS and OS of the mono- and poly-CC groups were compared (Fig. 1). The median RFS was not reached in the mono-CC HCC groups and 7.0 months in the poly-CC HCC groups. The RFS of patients with poly-CC HCC was significantly shorter than that of the mono-CC patients (Fig. 1a, p= 0.0084). The OS of patients with poly-CC tended to be shorter than that of patients with mono-CC, although the difference was not significant (Fig. 1b, p= 0.2966). Since poly-CC status appeared to be associated with the poorly differentiated HCC, we investigated the relationship with progenitor markers, cytokeratin 19 (CK19) and epithelial cell adhesion molecule (EpCAM). No significant difference of CK19 expressions was observed between mono- and poly-CC groups determined by immunohistochemistry. On the other hand, higher expression of EpCAM was observed in mono-CC than poly-CC significantly (*p* = 0.0304). Thus, CC status may not indicate the features of progenitor cells (Fig. S3).

**Table 2:** Clinicopathological features associated with clonal composition in surgical resected hepatocellular carcinoma

Variable	Category	CC = 0 (*n* = 8)	CC ≥ 1 (*n* = 26)	*p*
Sex	Male/female	6/2 (75.0/25.0)	15/11 (57.7/42.3)	0.4438
Age	< 70/ ≥ 70	3/5 (37.5/62.5)	11/15 (42.3/57.7)	1.0000
Liver function status	Child–Pugh A/B	7/1 (87.5/12.5)	24/2 (92.3/7.7)	1.0000
Etiology	HBV/HCV/alcoholic/normal	1/5/0/2 (12.5/62.5/0/25.0)	5/8/2/11 (19.2/30.8/7.7/42.3)	0.5036
ALT (U/l)	< 30/ ≥ 30	2/6 (25.0/75.0)	14/12 (53.8/46.2)	0.2327
Total bilirubin (mg/dl)	< 1/ ≥ 1	6/2 (75.0/25.0)	18/8 (69.2/30.8)	1.0000
Albumin (g/dl)	< 4/ ≥ 4	3/5 (37.5/62.5)	11/15 (42.3/57.7)	1.0000
ICGR15 (%)	< 15/ ≥ 15	5/3 (62.5/37.5)	15/11 (57.7/42.3)	1.0000
AFP (ng/ml)^a^	< 20/ ≥ 20	4/4 (50.0/50.0)	10/15 (40.0/60.0)	0.6951
PIVKA-II (mAU/ml)^a^	< 40/ ≥ 40	6/2 (75.0/25.0)	7/18 (28.0/72.0)	0.0351*
Tumor size (cm)	< 4.0/ ≥ 4.0	5/3 (62.5/37.5)	11/15 (42.3/57.7)	0.4290
Tumor number	Single/multiple	4/4 (50.0/50.0)	23/3 (88.5/11.5)	0.0374*
Microscopic vascular invasion	Positive/negative	4/4 (50.0/50.0)	24/2 (92.3/7.7)	0.0180*
Histologic grade^b^	Well/moderate, poor	4/3 (57.1/42.9)	4/21 (16.0/84.0)	0.0469*
Stage^c^	I/II/III/IVa	1/2/2/2 (14.3/28.6/28.6/28.6)	0/5/18/3 (0/19.2/69.2/11.5)	0.0819
History of previous treatment	Present/absent	4/4 (50.0/50.0)	3/23 (11.5/88.5)	0.0374*
Early recurrence	Yes/no	2/6 (25.0/75.0)	20/6 (76.9/23.1)	0.0127*

The asterisk indicates statistical significance

^a^Missing data in one patient with CC ≥ 1

^b^Unknown cases in one patient with CC = 0 and in one patient with CC ≥ 1

^c^Missing data in one patient with CC = 0

### Association of clonal composition status with somatic mutations

**Fig. 2:**
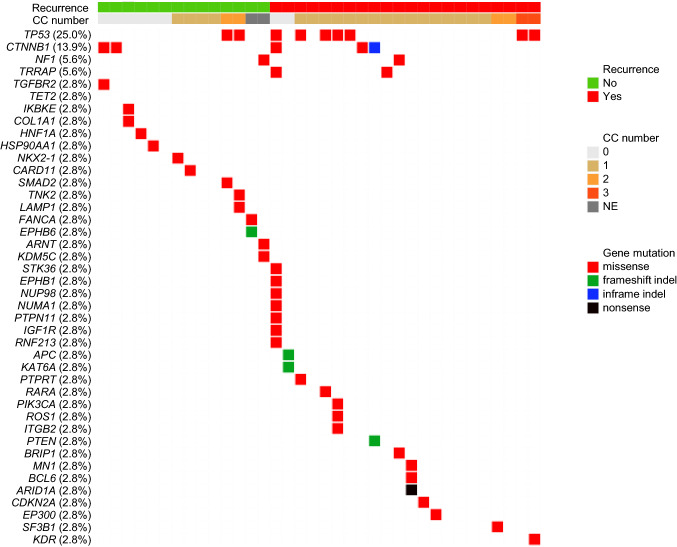
Profiling of pathogenic mutations detected in HCC tissue samples. The different mutation types, CC numbers, and cases with recurrences are color-coded as indicated in the figure

Somatic non-synonymous mutations in tissue samples (*n* = 36) were analyzed using targeted deep sequencing. Figure 2 summarizes the pathogenic variants with a FATHMM score ≥ 0.7 among the somatic variants. Pathogenic mutations of *TP53* (9/36, 25.0%) and *CTNNB1* (5/36, 13.9%) were frequently identified mutations seen in the 36 samples that were analyzed, as reported previously [21]. No significant association was seen between the presence of a pathogenic *TP53* gene mutation and grouping in the mono- and poly- CC groups (12.5% vs. 30.8%, *p* = 0.4030). Pathogenic *CTNNB1* mutations tended to occur frequently in the mono-CC group, but the difference was not significant (37.5% vs. 7.7%, *p* = 0.0721). The tumor mutation burden (TMB) has been reported as the total number of non-synonymous variants per tumor genomic region [22]. The TMB level of HCC is relatively low, compared with other types of solid cancers [23]. The TMB score of the presently reported cohort was also not high (median TMB = 6.7), and no difference in TMB was observed between the poly-CC (median 6.7; range 2.5–12.7) and the mono-CC (median 6.7; range 4.2–12.6) groups.

### Gene expression profile and functional enrichment analysis

**Fig. 3:**
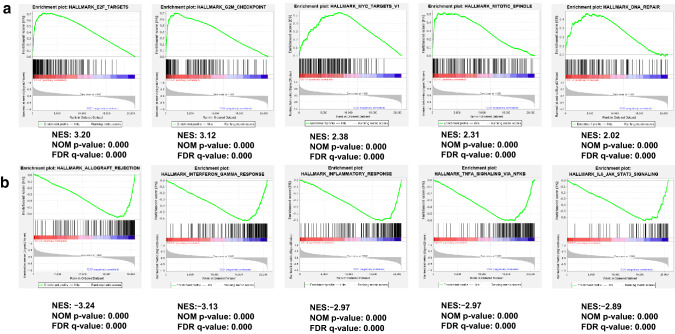
Enrichment plots of gene expression signatures for top five upregulated hallmark signatures (**a**) and downregulated hallmark signatures (**b**) pathways in poly-CC tumors. NES, normalized enrichment score; NOM p value, Nominal p value; FDR q value: false discovery rate

Thus far, our findings have indicated that the biological behavior of mono- and poly-CC tumors may differ. To investigate the biological functional differences between mono- and poly-CC tumors, we performed gene-set enrichment and pathway analysis using gene expression data from the Ion AmpliSeq Transcriptome Human Gene Expression assay. GSEA was performed for the enrichment of 50 hallmark gene sets from the MSigDB database. The enriched up- and down-regulated hallmarks with nominal *p* < 0.05 and a false discovery rate (FDR) with *q* < 0.05 are shown in Table 3. The top five enriched gene set in the poly-CC group were related to E2F targets, the G2M checkpoint, Myc targets, the mitotic spindle, and DNA repair (Fig. 3a). On the other hand, the top five down-regulated signatures in the poly-CC group were related Allograft rejection, IFNγ response, Inflammatory response, TNFα signaling via NFκB, and IL6-JAK-STAT3 signaling (Fig. 3b), indicating that the immune response is upregulated in mono-CC group.

**Table 3:** Gene set enrichment analysis (GSEA) results according to the MSigDB hallmark gene sets

	MSigDB hallmark pathway	NES	NOM *p* value	FDR *q* value
Upregulated hallmark signatures	E2F targets	3.20	0	0
	G2M checkpoint	3.12	0	0
	Myc targets v1	2.38	0	0
	Mitotic spindle	2.31	0	0
	DNA repair	2.02	0	0
	Myc targets v2	1.82	0	0.0003
	mTORC1 signaling	1.81	0	0.0004
	Spermatogenesis	1.69	0.0015	0.0020
	Oxidative phosphorylation	1.58	0	0.0077
	Unfolded protein response	1.58	0.0044	0.0069
	Protein secretion	1.52	0.0104	0.0136
	Cholesterol homeostasis	1.46	0.0162	0.0205
Downregulated hallmark signatures	Allograft rejection	− 3.24	0	0
	Interferon gamma response	− 3.13	0	0
	Inflammatory response	− 2.97	0	0
	TNFa signaling via NFKB	− 2.97	0	0
	IL6-JAK-STAT3 signaling	− 2.89	0	0
	Epithelial mesenchymal transition	− 2.75	0	0
	Interferon alpha response	− 2.57	0	0
	Complement	− 2.41	0	0
	IL2-STAT5 signaling	− 2.26	0	0
	Coagulation	− 2.15	0	0
	Apoptosis	− 2.03	0	0
	Angiogenesis	− 1.98	0.003	0
	KRAS signaling up	− 1.94	0	0
	Hypoxia	− 1.83	0	0.001
	UV response Dn	− 1.66	0	0.003
	Xenobiotic metabolism	− 1.66	0	0.003
	Myogenesis	− 1.37	0.003	0.043
	Apical surface	− 1.37	0.043	0.042

*NES* normalized enrichment score, *NOM* nominal, *FDR* false discovery rate

The top 100 up-regulated and 100 down-regulated genes (*p* < 0.05) in poly-CC group are listed in Table S1. Among the 100 up-regulated genes in poly-CC, the number of genes corresponding to the E2F target, G2M checkpoint, and mitotic spindle pathway were 19, 19, and 15, respectively. The top five genes with a higher fold change (FC) of up-regulation in poly-CC related were *BIRC5* (FC = 4.85, *p* = 0.0030), *MYBL2* (FC = 4.67, *p* = 0.0144), *CENPF* (FC = 4.03, *p* = 0.0007), *DLGAP5* (FC = 4.03, *p* = 0.0038), and *NEK2* (FC = 3.95, *p* = 0.0010) genes. These genes are members of gene sets related to E2F targets, G2M checkpoint, and mitotic spindle pathways. In addition, gene expression level of *MKI67*, marker of proliferation Ki-67, was significantly higher in poly-CC compared with mono-CC group (FC = 2.75, *p* = 0.0065). Among the 100 down-regulated genes in poly-CC, the number of genes corresponding to the IFNγ response, Inflammatory response, and IL6-JAK-STAT3 signaling were 3 (*CCL2*, *KLRK1*, and *HLA-DMA*), 4 (*CSF3R*, *PCSK9*, *IFNGR1*, and *HMOX1*), and 3 (*CSF3R*, *CCL2*, and *MARCO*), respectively. The analysis of immune infiltrates affecting CC status showed a significant correlation between mono-CC and infiltration of macrophages (Fig. S4).

Previous reports revealed the association between the expression of OATP1B3, which is encoded by the gene *SLCO1B3*, was strongly associated with Wnt/β-catenin signaling [28]. *SLCO1B3* gene expression was compared between mono-CC and poly-CC. Increased expression of *SLCO1B3* was observed in mono-CC (mean ± SD, 2.98 ± 2.08) compared with poly-CC (mean ± SD, 1.69 ± 2.24) (*p* = 0.1173). Although not significantly different, expression of *SLCO1B3* tended to be higher in mono-CC. In addition, expression of *SLCO1B3* was higher in the *CTNNB1* mutation positive cases (mean ± SD, 4.15 ± 2.73) than negative cases (mean ± SD, 1.62 ± 1.97) (*p* = 0.0691). This suggests that mono-CC may be associated with Wnt/β-catenin activated subtype HCC.

**Fig. 4:**
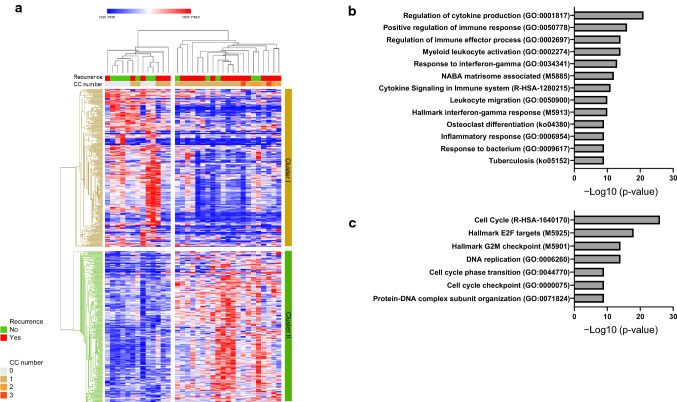
Hierarchical clustering analysis of differentially expressed genes. **a** Hierarchical clustering analysis of 250 upregulated and 250 downregulated genes in poly-CC tumors compared with mono-CC tumors. Clustering was based on average linkage and the one minus Pearson correlation distance using Morpheus, and two major clusters (clusters I and II) were formed. **b** Plot shows the top biological pathways enriched in cluster I from the GO Biological Processes, Hallmark gene sets, and KEGG database using Metascape. **c** Plot shows the top biological pathways enriched in cluster II from the GO Biological Processes, Hallmark gene sets, and KEGG database using Metascape

To further explore this notion, we analyzed significant gene sets in the mono- and poly-CC tumors. A hierarchical clustering analysis of 500 differentially expressed genes revealed the enrichment of cluster I and cluster II genes in mono- and poly-CC tumors, respectively (Fig. 4a). Early recurrent patients exhibited the enrichment of cluster II genes. We conducted a gene ontology (GO) analysis using Metascape and found significant enrichment of the cellular pathways associated with the immune response and cytokine-related pathways in cluster I, which was enriched in mono-CC tumors (Fig. 4b). On the other hand, cell cycle-related pathways including those related to E2F targets, the G2M checkpoint, and DNA replication were enriched in cluster II, which was enriched in poly-CC tumors (Fig. 4c).

### Univariate and multivariate analyses for risk factors of early recurrence

To identify the risk factors contributing to early postoperative recurrence of HCC, univariate and multivariate analysis were performed (Table 4). Univariate analysis showed that NBNC HCC, large tumor size, and elevated PIVKA-II level were significantly correlated with early recurrence. In multivariate analysis, large tumor size was found to be a significant risk factor; CC status was not significant, but there was a trend toward a higher risk of early recurrence in poly-CC (*p* = 0.0846).

**Table 4:** Prognostic factors for early recurrence in surgical resected hepatocellular carcinoma

Variable	Category	Early recurrence	No recurrence	Univariate	Multivariate
		(*n* = 22)	(*n* = 14)	*p*	*p*
Sex	Male/female	13/9 (59.1/40.9)	9/5 (64.3/35.7)	1.0000	–
Age	< 70/ ≥ 70	11/11 (50.0/50.0)	4/10 (28.6/71.4)	0.3021	–
Liver function status	Child–Pugh A/B	20/2 (90.9/9.1)	13/1 (92.9/7.1)	1.0000	–
HBV status	Positive/negative	2/20 (9.1/90.9)	5/9 (35.7/64.3)	0.0842	–
HCV status	Positive/negative	7/15 (31.8/68.2)	7/7 (50.0/50.0)	0.3142	–
NBNC	Yes/no	13/9 (59.1/40.9)	2/12 (14.3/85.7)	0.0142*	0.4458
ALT (U/l)	< 30/ ≥ 30	11/11(50.0/50.0)	7/7 (50.0/50.0)	1.0000	–
Total bilirubin (mg/dl)	< 1/ ≥ 1	14/8 (63.6/36.4)	11/3 (78.6/21.4)	0.4672	–
Albumin (g/dl)	< 4/ ≥ 4	9/13 (40.9/59.1)	5/9 (35.7/64.3)	1.0000	–
ICGR15 (%)	< 15/ ≥ 15	13/9 (59.1/40.9)	8/6 (57.1/42.9)	1.0000	–
AFP (ng/ml)^a^	< 20/ ≥ 20	8/13 (38.1/61.9)	7/7 (50.0/50.0)	0.5108	–
PIVKA-II (mAU/ml)^a^	< 40/ ≥ 40	4/17 (19.0/81.0)	10/4 (71.4/28.6)	0.0041*	0.2889
Tumor size (cm)	< 4.0/ ≥ 4.0	6/16 (27.3/72.7)	12/2 (85.7/14.3)	0.0016*	0.0433*
Tumor number	Single/multiple	19/3 (86.4/13.6)	10/4 (71.4/28.6)	0.3940	–
Microscopic vascular invasion	Positive/negative	20/2 (90.9/9.1)	10/4 (71.4/28.6)	0.1812	–
Histologic grade^b^	Well/moderate, poor	3/18 (14.3/85.7)	5/8 (38.5/61.5)	0.2106	–
Stage^c^	I, II/III, IVa	4/18 (18.2/81.8)	5/8 (38.5/61.5)	0.2427	–
History of previous treatment	Present/absent	3/19 (13.6/86.4)	4/10 (28.6/71.4)	0.3940	–
Clonal composition^d^	Poly-CC/mono-CC	20/2 (90.9/9.1)	6/6 (50.0/50.0)	0.0127*	0.0846

The asterisk indicates statistical significance

^a^Missing data in one patient with recurrence

^b^Unknown cases in one patient with recurrence and in one patient with non-recurrence

^c^Unknown cases in one patient with non-recurrence

^d^CC numbers could not be estimated in the two cases of patients without recurrence

## Discussion

Tumor heterogeneity is considered to be associated with a poor prognosis and outcome in cancer patients. In this study, we evaluated the CC number, a surrogate marker of intratumor heterogeneity, to investigate its clinical relevance in HCC. In general, HCC is reported to be highly heterogeneous [2–4]. In our sample set, the rate of homogeneous tumors with a CC number of 0 and the rate of heterogeneous tumors with a CC number of greater than 1 were 23.5% and 76.5%, respectively, suggesting that HCC was often a heterogeneous tumor in our cohort. Heterogeneous tumors with poly-CC had a significantly higher rate of early recurrence. In addition, patients with poly-CC tumors were significantly associated with a poor postsurgical RFS. This finding suggests that HCC with high heterogeneity has a higher risk of recurrence after surgery. The CC number may be useful for predicting the risk of recurrence, but further studies are essential.

A gene mutation analysis was performed on the same sample set, and pathogenic mutations were selected using the FATHMM score. Therefore, the frequencies of *TP53* and *CTNNB1* mutations were lower than previously reported [21]. The gene mutation analysis showed no association between the CC number and the gene mutations status, including the TMB. The calculation of CC number is based on the copy number loss, suggesting that the diversity of copy number variations is reflected by the CC number. In lung cancer, subclonal evolution based on copy number changes is reportedly associated with the RFS and OS to a greater degree than those based on single nucleotide mutations [24]. Evaluation of the CC number based on copy number loss may be more informative for predicting clinical outcome than the detection of single nucleotide variants in hepatocellular cancer.

In an enriched transcriptome analysis using a supervised approach, cell cycle-related pathways were among the top 5 upregulated pathways in poly-CC tumors. Oshi et al. showed that G2/M scoring is associated with cell proliferation-related factors and the breast cancer grade using an enrichment analysis based on a gene expression analysis of metastatic estrogen-receptor-positive breast cancer [25]. The pathways enriched in our cohort were related to the entire cell cycle progression in the G1, S and G2/M phases, suggesting active involvement in cell cycle progression and proliferation. The *BIRC5*, which was common to all three pathways and showed the highest difference, plays important rules in the E2F, G2M, and mitosis pathways, highlighting the importance of *BIRC5* involvement in poly-CC tumor, particularly with regards to enhanced functions in cell cycle progression, division, and proliferation [21]. The other four genes have also been reported to be involved in proliferation in hepatocellular carcinoma [22–25], suggesting that these genes could be related to the increased proliferative phenotype of poly-CC. Recently, Llovet et al. [26]. reviewed the molecular classification of HCC that classifies HCC into “proliferation” and “non-proliferation” classes based on a previous transcriptome analysis [27]. The proliferation class is characterized by enriched pathways related to the cell cycle, *TP53* mutation, and HBV-positive populations. Compared with the mono-CC group, the poly-CC group in our sample cohort tended to exhibit enriched E2F and other cell cycle pathways, a high *TP53* mutation rate (8/26 [30.8%] vs. 1/8 [12.5%]), and HBV positivity (5/26 [19.2%] vs. 1/8 [12.5%]), although these differences were not significant because of the small sample size. Thus, poly-CC tumors (cluster II) are consistent with these molecular and etiological characteristics of the “proliferative class.” On the other hand, the non-proliferation class is associated with HCV and a molecular profile characterized by enhanced IL6-JAK-STAT signaling or *CTNNB1* mutation-mediated Wnt/β-catenin signaling associated with inflamed pathogenicity [28–30]. Mono-CC tumors, compared with poly-CC tumors, are characterized by non-HBV (7/8 [87.5%] vs. 21/26 [80.8%]), *CTNNB1* mutation (3/8 [37.5%] vs. 2/26 [7.7%]), and enriched cytokine-related pathways. The analysis of immune infiltrates affecting CC status showed a significant correlation between mono-CC and infiltration of macrophages (Fig. S4). This result might be linked to the enrichment of IFNγ- and IL-6-mediated immune response pathway in mono-CC [31, 32]. Thus, mono-CC tumors (cluster I) are consistent with these molecular and etiological characteristics of the “non-proliferative class”.

Tumor heterogeneity of HCC may be different between tumor tissue of different etiology because of the disease features and the period of fibrogenesis as well the extent of chromosomal mutations and this phenomena is exemplified in this study which includes a heterogenous population HCC patients with different etiologies. The limitations of this study include its retrospective design and small sample size. Future studies examining larger sample sizes are needed to confirm the findings of this study and differential signatures of HCC with various etiologies. In the present study, pathway-related analysis was performed to characterize poly-CC tumors. However, the further analysis will be planned in the next study. In addition, isolation and transplantation of different poly-CC HCC cells in animals may elucidate copy number relevance in infiltration and proliferation rate.

In conclusion, HCCs with poly-CCs, a surrogate of heterogeneity based on copy number variation, are enriched in genes involved in the cell cycle pathway, have a proliferative phenotype, and have a higher risk of early postoperative recurrence.

## Supplementary Information

Below is the link to the electronic supplementary material.

Supplementary file1 Fig. S1. Representative whole-genome view of HCC tumors. The distribution of whole-genome copy number (PDF 221 KB)

Supplementary file2 Fig. S2. Representative analysis of clonal composition number. The plots in each left panel were derived from HCC samples with a CC number of 3 (A), 2 (B), or 1 (C). The x-axis depicts the log2R of BAF, and the y-axis the log2R for the copy number. The size of each symbol reflects the copy number, the percentage of aberrant tumor cells (%AC) is differentiated by color, and the number of minor alleles at a heterozygous site (NOMA) is indicated by the different symbols (circle, cross, diamond, and square for values of 0, 1, 2, and 3, respectively). The CC number was estimated within regions where NOMA=0. Points with the same %AC correspond to the same clone. The right panels show the graphical representations of log2R and BAF for each aberrant segment indicated by the numbered yellow circles in each left panel. The arrows indicate clones (PDF 302 KB)

Supplementary file3 Fig. S3. Immunohistochemical (IHC) analysis of progenitor marker protein. Representative photomicrographs of a tumor showing negative IHC expression of EPCAM (A) and CK19 (D). Representative photomicrograph of a tumor showing a heterogenous EpCAM (B) and CK19 staining pattern (E). Red outlines correspond to high magnification photomicrographs. Re Low magnification scale bar = 1000 µm and high magnification scale bar = 200 µm. Plots of H scores for EpCAM (C) and CK19 (F) in cancer cells. The p value was calculated by Mann-Whitney U test. ns, not significant (PDF 415 KB)

Supplementary file4 Fig. S4. Violin plot showing the infiltration levels of six types of immune cells estimated by TIMER2.0. The p value was calculated by two-tailed Student's t test. ns, not significant (PDF 224 KB)

Supplementary file5 (XLSX 53 KB)

## Data Availability

Yes. Data generated or analyzed during this study are available from the corresponding author on reasonable request.

## Abbreviations

AFP: Alpha-fetoprotein
ALT: Alanine aminotransferase
BAF: B-allele frequency
CC: Clonal composition
CCP: Comprehensive cancer panel
FATHMM: Functional analysis through hidden Markov models
FC: Fold change
FDR: False discovery rate
FFPE: Formalin-fixed, paraffin-embedded
GO: Gene ontology
GSEA: Gene set enrichment analysis
HBV: Hepatitis B virus
HCC: Hepatocellular carcinoma
HCV: Hepatitis C virus
ICGR15: Indocyanine green retention rate at 15 min
IFNγ: Interferon gamma
IGV: Integrative genomics viewer
IL6: Interleukin-6
JAK: Janus-associated kinase
MIP: Molecular inversion prove
MOMA: Number of minor alleles
MSigDB: Molecular signatures database
NBNC: Non-hepatitis B and non-hepatitis C virus
NFκB: Nuclear factor-kappa B
OS: Overall survival
PIVKA-II: Protein induced by vitamin K absence or antagonist-II
PTPE: Percutaneous transhepatic portal vein embolization
RFA: Radiofrequency ablation
RFS: Recurrence-free survival
STAT3: Signal transducer and activator of transcription 3
TACE: Transcatheter arterial chemoembolization
TMB: Tumor mutation burden
TNFα: Tumor necrosis factor alpha
