# Supplementary figures and images for: Patients with polyclonal hepatocellular carcinoma are at a high risk of early recurrence and have a poor recurrence-free survival period
Source: Hepatol Int. 2022 Jan 1;16(1):135–47. doi: 10.1007/s12072-021-10278-4 (PMC8843910; doi:10.1007/s12072-021-10278-4)

Figure S1

A

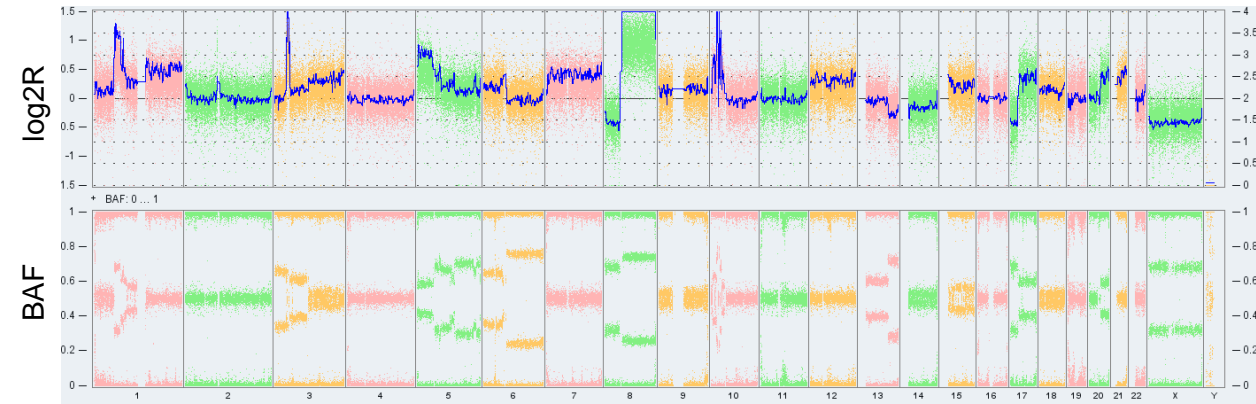

B

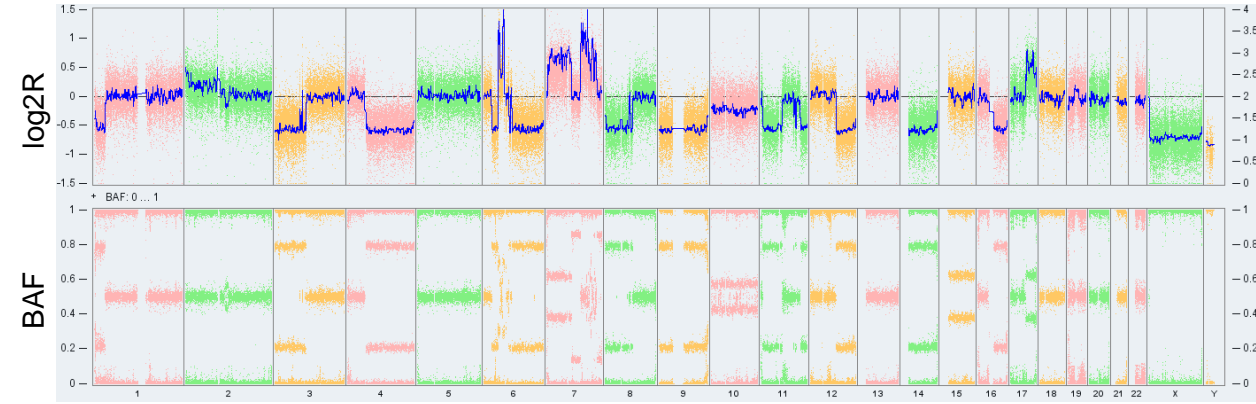

C

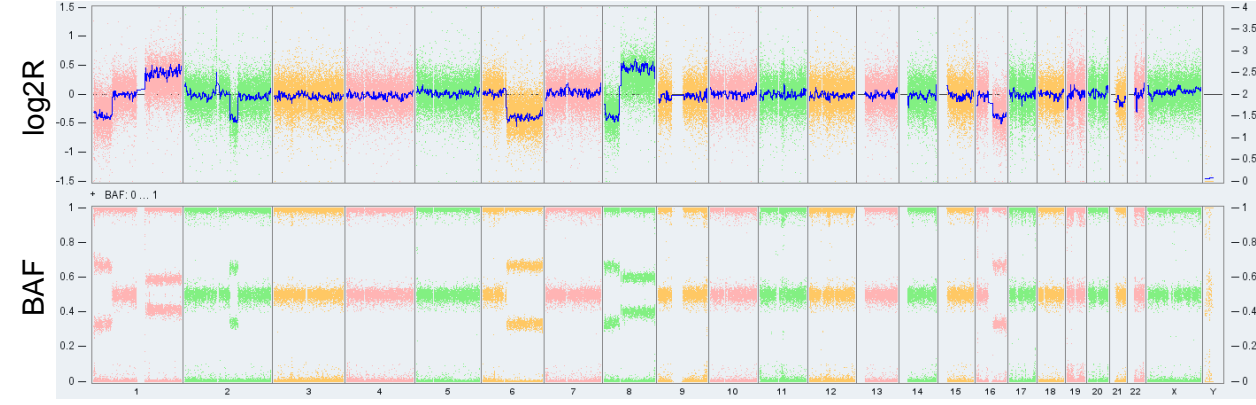

Supplement: Supplementary file 1 — Supplementary file1 Fig. S1. Representative whole-genome view of HCC tumors. The distribution of whole-genome copy number (PDF 221 KB) [file 12072_2021_10278_MOESM1_ESM.pdf]

Figure S2 A

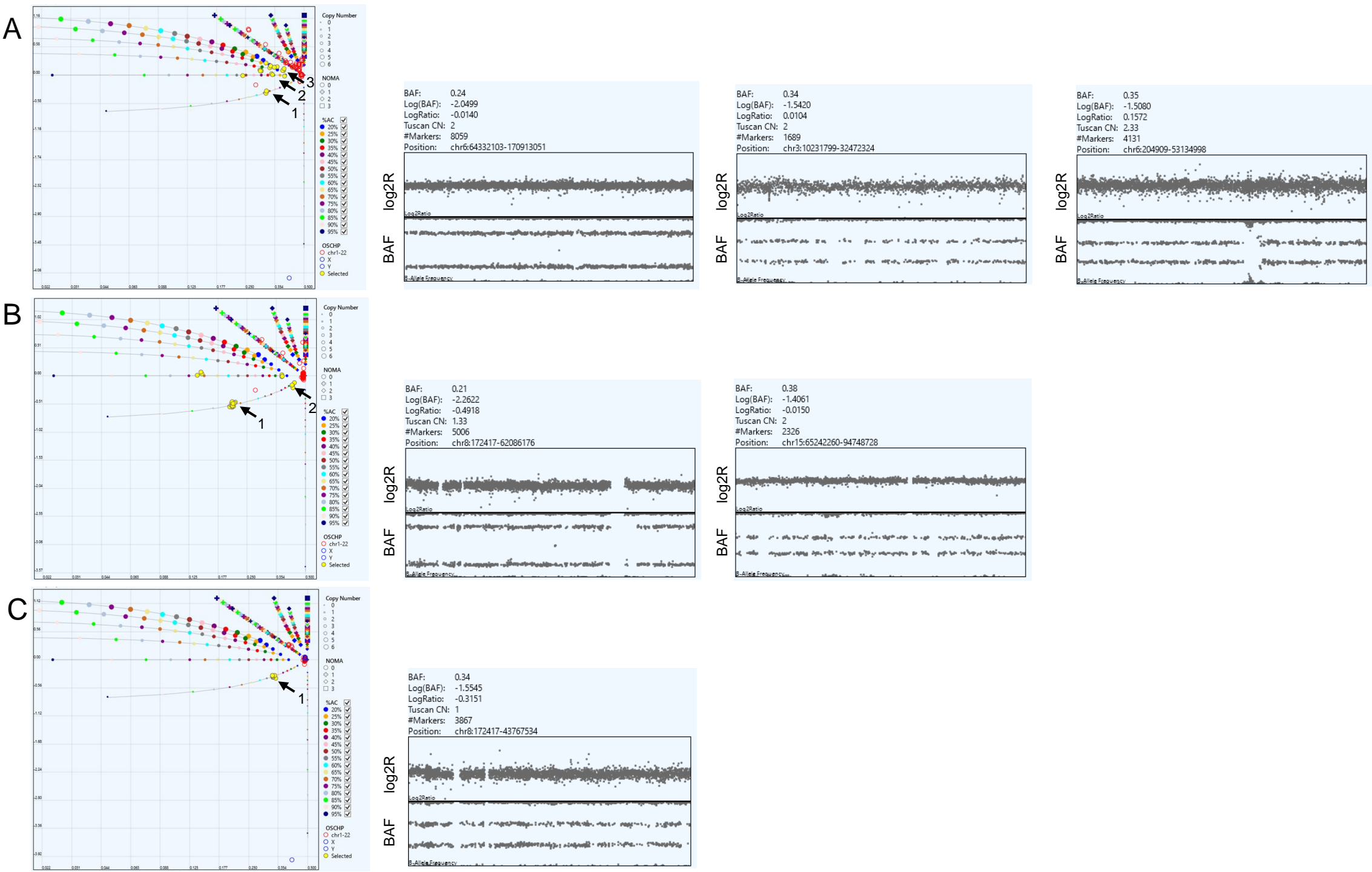

Supplement: Supplementary file 2 — Supplementary file2 Fig. S2. Representative analysis of clonal composition number. The plots in each left panel were derived from HCC samples with a CC number of 3 (A), 2 (B), or 1 (C). The x-axis depicts the log2R of BAF, and the y-axis the log2R for the copy number. The size of each symbol reflects the copy number, the percentage of aberrant tumor cells (%AC) is differentiated by color, and the number of minor alleles at a heterozygous site (NOMA) is indicated by the different symbols (circle, cross, diamond, and square for values of 0, 1, 2, and 3, respectively). The CC number was estimated within regions where NOMA=0. Points with the same %AC correspond to the same clone. The right panels show the graphical representations of log2R and BAF for each aberrant segment indicated by the numbered yellow circles in each left panel. The arrows indicate clones (PDF 302 KB) [file 12072_2021_10278_MOESM2_ESM.pdf]

## Figure S3

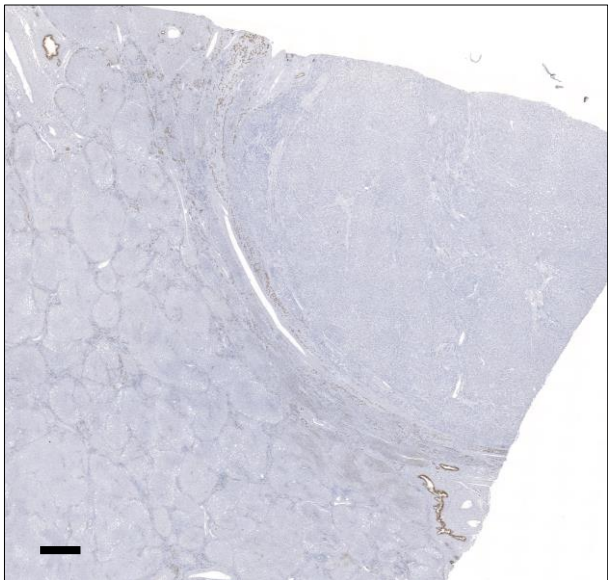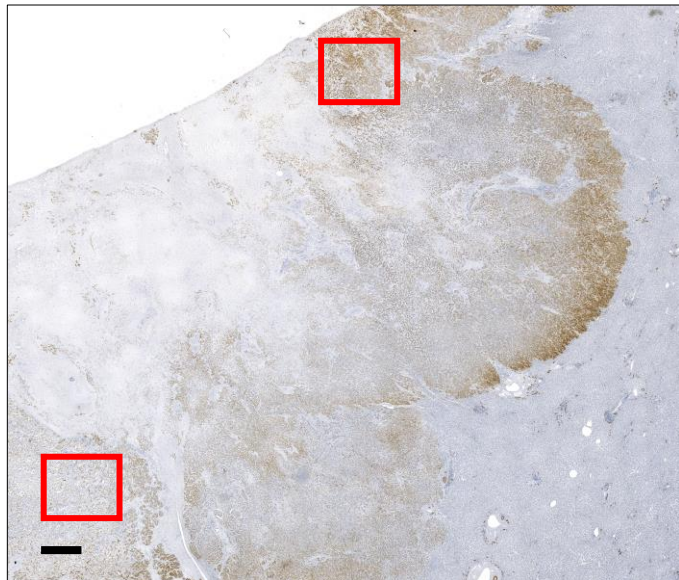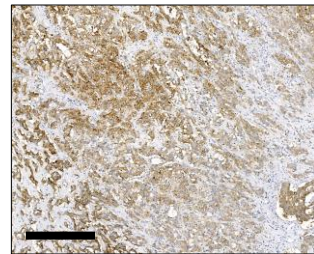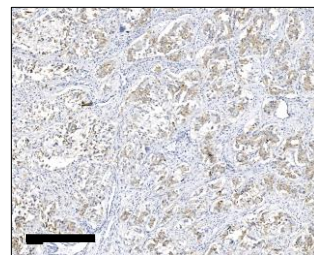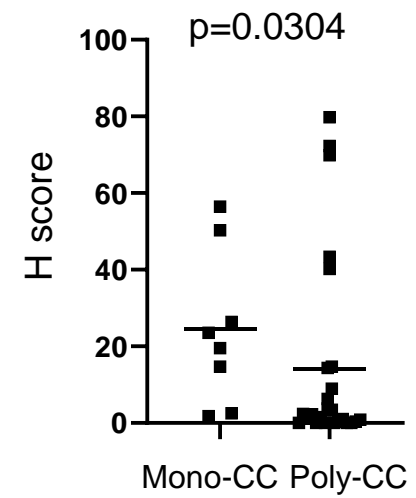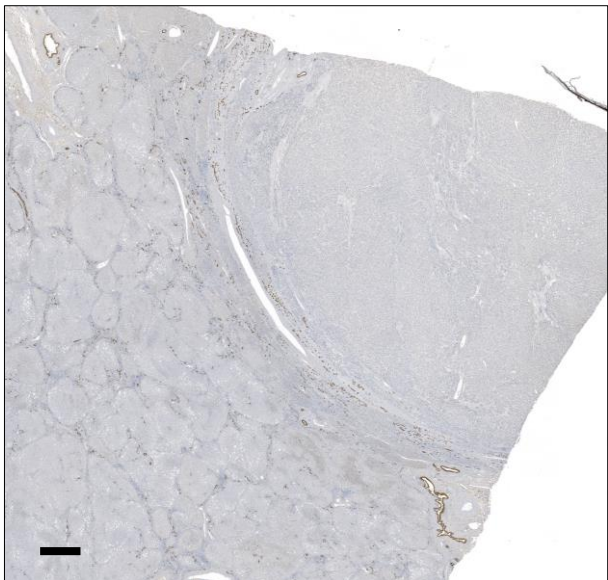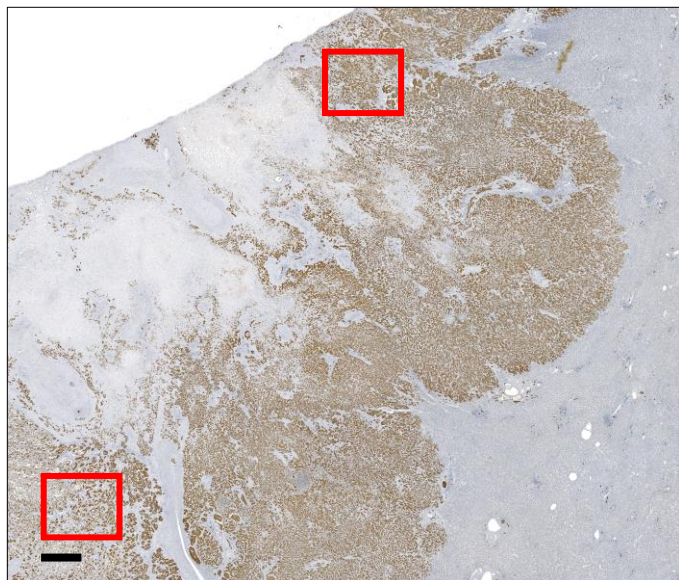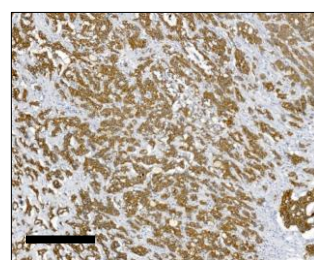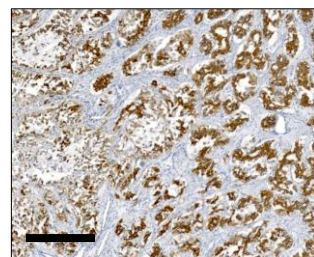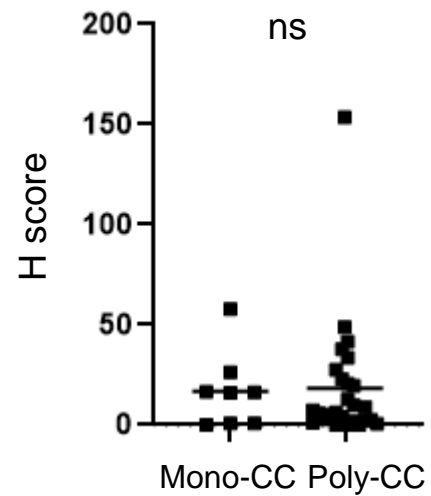

Supplement: Supplementary file 3 — Supplementary file3 Fig. S3. Immunohistochemical (IHC) analysis of progenitor marker protein. Representative photomicrographs of a tumor showing negative IHC expression of EPCAM (A) and CK19 (D). Representative photomicrograph of a tumor showing a heterogenous EpCAM (B) and CK19 staining pattern (E). Red outlines correspond to high magnification photomicrographs. Re Low magnification scale bar = 1000 µm and high magnification scale bar = 200 µm. Plots of H scores for EpCAM (C) and CK19 (F) in cancer cells. The p value was calculated by Mann-Whitney U test. ns, not significant (PDF 415 KB) [file 12072_2021_10278_MOESM3_ESM.pdf]

Figure S4

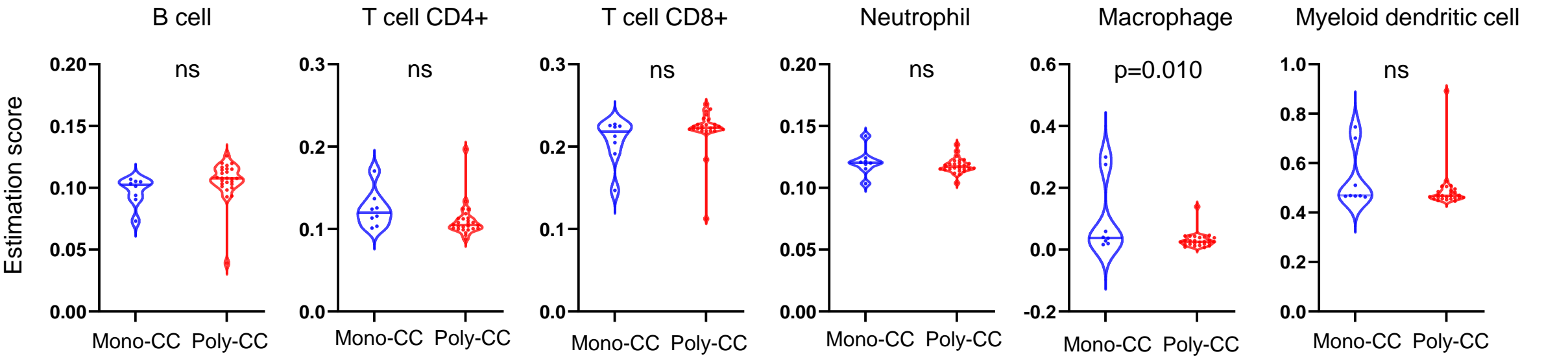

Supplement: Supplementary file 4 — Supplementary file4 Fig. S4. Violin plot showing the infiltration levels of six types of immune cells estimated by TIMER2.0. The p value was calculated by two-tailed Student's t test. ns, not significant (PDF 224 KB) [file 12072_2021_10278_MOESM4_ESM.pdf]
